# Rapid Preparation of Superabsorbent Self-Healing Hydrogels by Frontal Polymerization

**DOI:** 10.3390/gels9050380

**Published:** 2023-05-05

**Authors:** Ying Qin, Hao Li, Hai-Xia Shen, Cai-Feng Wang, Su Chen

**Affiliations:** State Key Laboratory of Materials-Oriented Chemical Engineering, College of Chemical Engineering, Nanjing Tech University, 5 Xin Mofan Road, Nanjing 210009, China; qinying717@163.com (Y.Q.); leo541437118@163.com (H.L.); shx@njtech.edu.cn (H.-X.S.)

**Keywords:** frontal polymerization, hydrogels, porous morphology, swelling behavior, self-healing

## Abstract

Hydrogels have received increasing interest owing to their excellent physicochemical properties and wide applications. In this paper, we report the rapid fabrication of new hydrogels possessing a super water swelling capacity and self-healing ability using a fast, energy-efficient, and convenient method of frontal polymerization (FP). Self-sustained copolymerization of acrylamide (AM), 3-[Dimethyl-[2-(2-methylprop-2-enoyloxy)ethyl]azaniumyl]propane-1-sulfonate (SBMA), and acrylic acid (AA) within 10 min via FP yielded highly transparent and stretchable poly(AM-*co*-SBMA-*co*-AA) hydrogels. Thermogravimetric analysis and Fourier transform infrared spectroscopy confirmed the successful fabrication of poly(AM-*co*-SBMA-*co*-AA) hydrogels with a single copolymer composition without branched polymers. The effect of monomer ratio on FP features as well as porous morphology, swelling behavior, and self-healing performance of the hydrogels were systematically investigated, showing that the properties of the hydrogels could be tuned by adjusting the chemical composition. The resulting hydrogels were superabsorbent and sensitive to pH, exhibiting a high swelling ratio of up to 11,802% in water and 13,588% in an alkaline environment. The rheological data revealed a stable gel network. These hydrogels also had a favorable self-healing ability with a healing efficiency of up to 95%. This work contributes a simple and efficient method for the rapid preparation of superabsorbent and self-healing hydrogels.

## 1. Introduction

Hydrogels are soft materials with three-dimensional cross-linked network structures and are able to swell in water without dissolving themselves [1,2,3]. The networks of hydrogels are established by covalent bonding or non-covalent interactions like physical entanglement, hydrogen bonding, ligand interactions, hydrophobic interactions, or electrostatic interactions [4,5,6,7]. A lot of natural polymers (for example, collagen, chitosan, and peptides) and synthetic polymers (for example, polyacrylamide and polyvinyl alcohol) have been extensively designed to produce hydrogels with fantastic qualities [8,9,10,11,12]. Through appropriate component design, preparation parameter optimization, and microstructure regulation, the properties of hydrogels like durability, ionic conductivity, hydrophilicity and hydrophobicity, flexibility, shape memory capacity, adhesion, and mechanical properties, can be tuned to meet the requirements of applications in a variety of fields, including biomedical applications, medicine, tissue engineering, environmental engineering, and agriculture [13,14,15,16]. Specifically, hydrogels composed of polymer networks with good hydrophilicity and flexibility can possess excellent water absorption and retention capacities, which could be useful as water-retaining agents in daily life and water management in sustainable agriculture and the environment [17]. The reticular structure of hydrogels similar to extracellular matrix could be beneficial for wound dressings, as such materials could absorb wound effusion whilst maintaining a moist environment facilitating tissue regeneration [9]. Additionally, hydrogels endowed with a biomimetic self-healing capacity can repair themselves spontaneously after damage to improve their application durability [18]. Despite great advances, the rapid and energy-efficient development of hydrogels with robust properties is still highly desirable.

As an attractive self-sustained preparation strategy, frontal polymerization (FP) rapidly converts monomers to polymers based on the heat released from the exothermic reaction itself, possessing several advantages including a fast reaction speed, low manufacturing energy consumption, ease of operation, and high product homogeneity [19,20,21]. In this polymerization system, the self-sustained reaction starts after a triggering event, allowing the appearance of a polymerization front that can rapidly self-propagate to accomplish the whole reaction without further energy input [22,23]. The conception of FP was first introduced in the 1970s, and developed by Russian chemists and Pojman [24,25]. Then, FP was developed to prepare various materials including resins, coatings, and foams, as well as functional hydrogels [22,26,27,28]. In particular, our group developed diverse FP modes to prepare a number of functional hydrogels, such as fluorescent nanocomposite hydrogels, biocompatible hydrogels, stimuli-responsive hydrogels, hydrogel actuators, or self-healing hydrogels [29,30,31,32,33,34,35,36,37,38,39]. Here, we explored new hydrogels with robust properties using the simple and efficient method of FP.

Molecular designs for a hydrogel system involve a trade-off between desirable properties. The development of hydrogels that have the ability to repair themselves after sustaining damage continues to be a significant scientific endeavor, since these materials could have prolonged lifespan and durability, restore their original properties, and show great promise in various areas such as tissue engineering, wound healing, soft robotics, and environmental remediation [5,13,32,40,41,42,43,44,45]. The self-healing ability depends mainly on dynamic cross-linking mechanisms, including reversible covalent bonds (e.g., borate ester bonds, disulfide bonds, and imine bonds) and non-covalent interactions (e.g., hydrogen bonds, ionic bonds, host–guest interactions and hydrophobic interactions) [6,41], whereas polymer network structure, crosslinking density, and intermolecular interactions contribute to the mechanical properties of hydrogels including strength, stiffness, toughness, elasticity, and stretchability [46,47,48,49,50]. For instance, hydrogels with extensible polymer networks from a high degree of polymerization may exhibit good stretchability due to the flexibility and mobility of the polymer chains [51]. However, there exists the incompatibility between desirable mechanical performance and self-healing for hydrogels, and self-healing hydrogels are usually mechanically weak and may collapse their network when subjected to deformation [7]. In this work, we performed copolymerization of acrylamide (AM), 3-((2-(methacryloyloxy)ethyl) dimethylammonium)propane-1-sulfonate (SBMA), and acrylic acid (AA) in the presence of crosslinkers, allowing the formation of robust flexible polymer networks for hydrogels. Strong non-covalent interactions including hydrogen bonding and electrostatic interactions form owing to the existence of abundant amide, carboxylic acid, and ester functional groups as well as −N^+^−CH_3_ and −SO_3_^−^ groups, which further reinforce the hydrogel network whilst guaranteeing the self-healing behavior of the hydrogels. The robust polymer network with rich hydrophilic groups could also make the hydrogels greatly swell in aqueous media without destroying the network structure and dissolving themselves.

Superabsorbent and self-healing hydrogels were rapidly synthesized from AM, SBMA, and AA via FP. The polymerization process was self-sustained and fast, finishing within 10 min, to produce highly transparent poly(AM-*co*-SBMA-*co*-AA) hydrogels. The FP features including frontal velocity and temperature distribution during the synthetic procedure, as well as the composition, structure, porous morphology, swelling behavior, rheological properties, and the self-healing capacity for the hydrogels were studied. The dependence of these properties on chemical composition (monomer ratio) for the hydrogels was revealed. The super swelling and self-healing mechanisms are discussed. The results provide insight into the easy preparation of new hydrogels with excellent swelling capacities and self-healing abilities in a time-saving, easily operated, and energy efficient route.

## 2. Results and Discussion

### 2.1. Preparation of Poly(AM-co-AA-co-SBMA) Hydrogels

**Figure 1:**
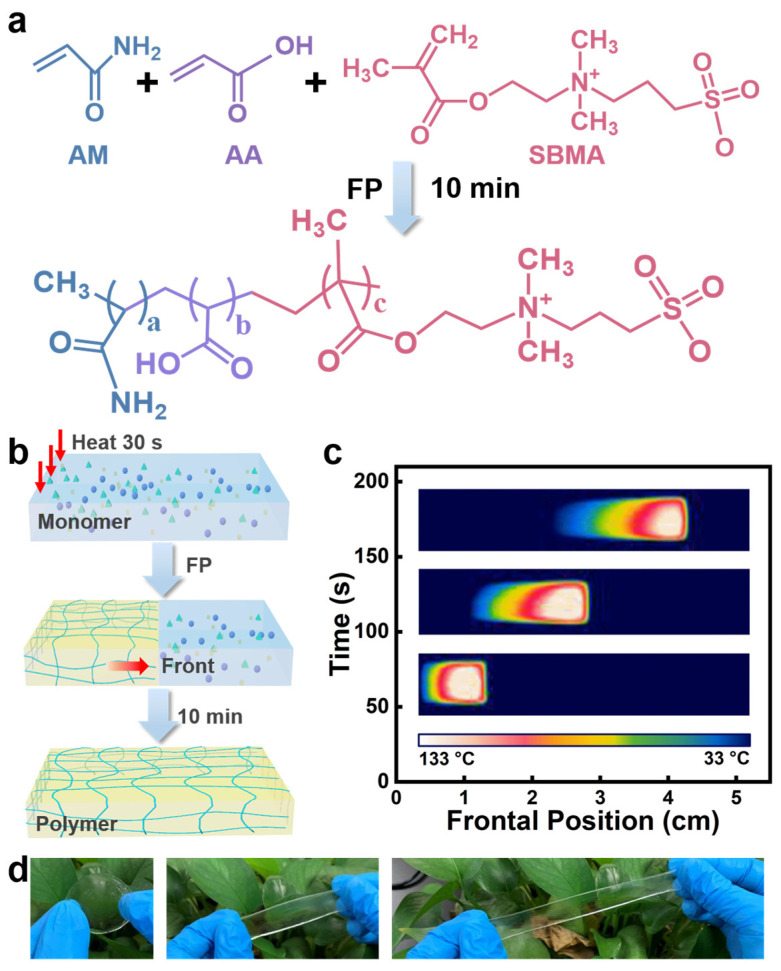
(**a**) The schematic diagram of poly(AM-*co*-AA-*co*-SBMA) hydrogel synthesis via FP. (**b**) The diagram illustration of the hydrogel fabrication route by a horizontal FP mode. (**c**) Infrared thermal image showing the propagation of the polymerization front over time during the preparation process of poly(AM-*co*-AA-*co*-SBMA) hydrogels. (**d**) Photographs of a sample of poly(AM-*co*-AA-*co*-SBMA) hydrogels under stretching.

In this work, superabsorbent and self-healing hydrogels were rapidly prepared from monomers of AM, AA, and SBMA within 10 min via horizontal FP (Figure 1a). AM and AA are ideal monomers for FP because both monomers have a high reactivity to implement exothermal polymerization which could provide sufficient heat energy for the self-sustained reaction mode, and the resulting polymers usually have favorable flexibility and mechanical properties [34,35]. The amphoteric ion SBMA is present in plants and has the advantages of good biocompatibility, water retention, and a low level of immunogenicity [52,53,54]. Copolymerization of these three monomers can also provide hydrophilic properties due to the abundance of hydrophilic groups and at the same time self-healing properties due to the abundance of molecular interactions between these functional groups, including hydrogen bonding between amide, carboxylic acid, and ester functional groups, as well as electrostatic interactions between −N^+^−CH_3_ and −SO_3_^−^ functional groups. Figure 1b shows the synthesis schematic of poly(AM-*co*-AA-*co*-SBMA) hydrogels in the horizontal FP reaction mode, and a picture of the experimental setup for preparing hydrogels by horizontal FP is shown in Figure S1a. The local heat for 30 s on one side of the reaction system of AM, AA, and SBMA with N,N″-bis(acryloyl)cystamine (BACA) as the cross-linker and ammonium persulfate (APS) in combination with N, N, N′, N′-tetramethylethylenediamine (TMEDA) as initiators, trigger the exothermal radical polymerization to form a polymerization front. The internal heat sources are removed when the polymerization front forms as the front self-propagates along the reactor using the exothermic reaction’s own heat energy (Figure 1c). Figure S1b shows photographs of the propagating front during FP. This process enables the complete transformation of monomers to hydrogel polymers. As such, poly(AM-*co*-AA-*co*-SBMA) hydrogels that are transparent and stretchable have been rapidly synthesized (Figure 1d).

**Figure 2:**
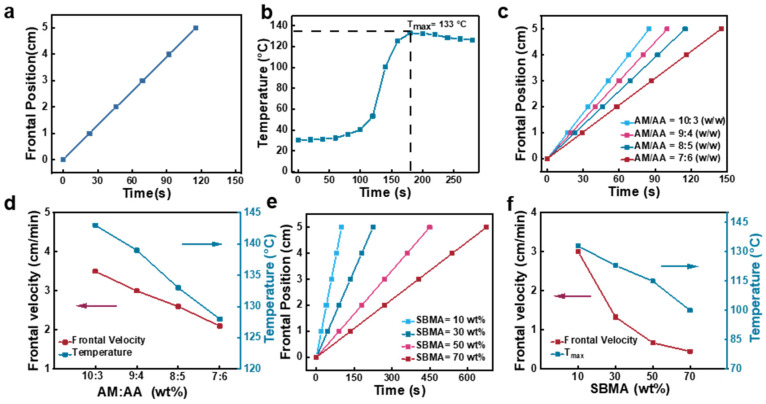
(**a**) Plot of frontal position versus reaction time in FP of poly(AM-*co*-AA-*co*-SBMA) hydrogels prepared with AM/AA = 8:5 (wt/wt) and SBMA = 10 wt%. (**b**) The dependence of temperature on reaction time, tested at a fixed point of 4.7 cm from the initial triggering point. (**c**) Frontal position vs. time curves and (**d**) the corresponding frontal velocities and *T*_max_ values during FP at different AM/AA mass ratios with 10 wt% SBMA. (**e**) Frontal position vs. time curves and (**f**) the corresponding frontal velocities and *T*_max_ values during FP at different SBMA mass concentrations with AM/AA mass ratio of 8:5 wt/wt.

The FP process for poly(AM-*co*-AA-*co*-SBMA) hydrogels was analyzed. We studied the frontal position (i.e., the position of the polymerization front) and temperature distribution over time, to ascertain whether the reaction system contains pure FP without spontaneously polymerization. Firstly, pure FP is characterized by a constant frontal velocity [25]. To this end, we employed an infrared thermal camera to confirm that a stable front formed during the synthesis of the hydrogel, which moved rapidly from left to right over time (Figure 1c). Based on this, we measured the frontal position over time and found that the plots of frontal position versus reaction time were linear (Figure 2a). According to the results, the frontal velocity remained constant, revealing pure FP in the poly(AM-*co*-AA-*co*-SBMA) hydrogel preparation without spontaneous polymerization. Figure 2b shows the typical temperature distribution during FP, wherein the presence of horizontally defined areas as well as a maximum temperature (*T*_max_) are more strong evidence of pure FP.

FP is highly dependent on the monomer ratio used for poly(AM-*co*-AA-*co*-SBMA) hydrogels. Frontal velocities and *T*_max_ are reliant on various monomer ratios of AM to AA and different SBMA concentrations were thoroughly investigated. Different AM/AA mass ratios of 10:3, 9:4, 8:5, and 7:6 wt/wt were selected to carry out FP. We found that the FP process was sustained for the whole reaction for all the above recipes, to obtain an array of hydrogels. The frontal position versus reaction time curves for the reaction systems containing various AM/AA mass ratios are displayed in Figure 2c. All of the plots showed linear relationships indicating stable frontal velocities and suggesting pure FP in all four polymerization systems. The corresponding frontal velocities and *T*_max_ values are shown in Figure 2d. When the AM/AA mass ratio decreased from 10:3 to 7:6 wt/wt, the frontal velocity declined from 3.5 to 2.1 cm/min and the corresponding *T*_max_ decreased from 143 to 128 °C. These results indicate that AM is more reactive than AA for the free radical polymerization reaction, and hence decreasing the AM concentration leads to a decline in both the frontal velocity and *T*_max_.

Figure 2e,f show the effect of the SBMA concentration on frontal velocity and *T*_max_: both showed a decreasing trend with an increase in the SBMA content. Specifically, when the mass ratio of SBMA increased from 10 wt% to 70 wt%, its frontal velocity decreased from 3 cm/min to 0.45 cm/min, and *T*_max_ decreased from 133 to 100 °C. These results revealed that an increase in SBMA causes a decrease in the release of the reaction heat, leading to a decrease in the frontal velocity, which further prolongs the heat loss and thus decreases *T*_max_.

### 2.2. Characterizations of Poly(AM-co-AA-co-SBMA) Hydrogels

**Figure 3:**
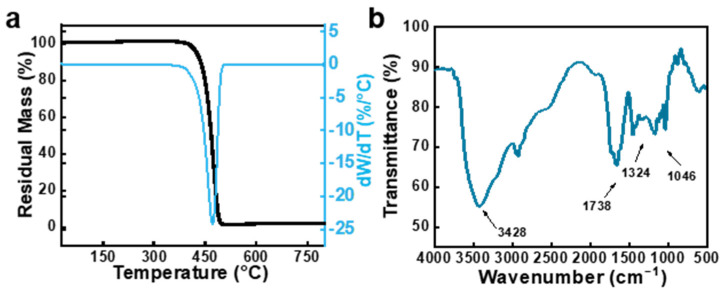
(**a**) Thermogravimetric (TG) curve and the corresponding DTG curve of poly(AM-*co*-AA-*co*-SBMA) hydrogels. (**b**) FT-IR spectroscopy of poly(AM-*co*-AA-*co*-SBMA) hydrogels. The hydrogels were prepared using the following composition: AM/AA = 8:5 (wt/wt), SBMA = 10 wt%, BACA = 0.5 wt%, APS = 0.2 wt%, glycol = 50 wt%, [APS]/[TMEDA] = 1:4 (mol/mol).

To completely remove unreacted monomers, poly(AM-*co*-AA-*co*-SBMA) hydrogels prepared via FP were submerged in deionized water that was changed every day for a week. Figure 3a shows the thermogravimetric (TG) curve of the hydrogels under a heating rate of 10 °C/min along with the differential thermogravimetric (DTG) curve that was obtained from the first-order differential of the matching TG. From the pyrolysis process, it can be seen that the backbone of the hydrogel polymer started to break and decompose at temperatures above 396 °C, showed a single degradation step between 396 and 510 °C, and had a residual mass of 1.63% of the original product’s mass. From these results, it is evident that poly(AM-*co*-AA-*co*-SBMA) hydrogels only have one degradation step, demonstrating the composition of the single copolymer. The results also confirmed the complete removal of unreacted monomers as well as the absence of other branched polymers in this reaction system. Then, Fourier transform infrared (FT-IR) was employed to analyze the structure of the poly(AM-*co*-AA-*co*-SBMA) hydrogels. As shown in Figure 3b, the typical peak observed at 1046 cm^−1^ was formed by the symmetric stretching vibration of the −SO_3_^−^ sulfonic acid group, the stretching vibrations of the quaternary –N^+^−CH_3_, C = O, and –NH_2_/−OH groups were represented by the typical absorption peaks at 1324, 1738, and 3428 cm^−1^, respectively. The above results demonstrate the successful copolymerization of AM, SBMA, and AA into the single copolymer poly(AM-*co*-AA-*co*-SBMA) by FP.

### 2.3. Morphology of Poly(AM-co-AA-co-SBMA) Hydrogels

**Figure 4:**
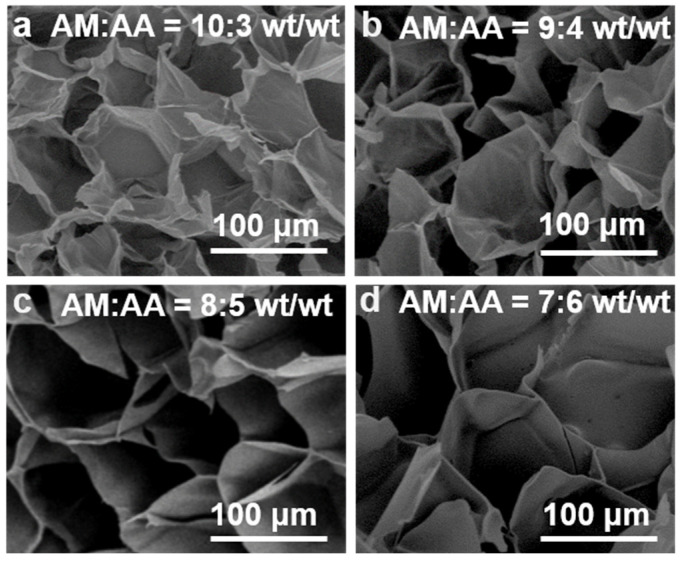
SEM micrographs of poly(AM-*co*-AA-*co*-SBMA) hydrogels synthesized using AM/AA ratios of (**a**) 10:3, (**b**) 9:4, (**c**) 8:5, and (**d**) 7:6 (wt/wt). SBMA = 10 wt%.

Scanning electron microscopy (SEM) images of poly(AM-*co*-AA-*co*-SBMA) hydrogels were measured to evaluate their morphology. As shown in Figure 4a–d, these prepared hydrogels had densely arranged microporous structures with a relatively narrow pore size distribution. Figure S2 shows the pore size distributions of the hydrogels. Specifically, the average pore sizes of the hydrogels were about 50, 88, 150, and 234 μm for the samples prepared with AM/AA = 10:3, 9:4, 8:5, and 7:6 wt/wt, respectively. As the AM/AA mass ratio decreased, the pore size of the resulting hydrogels increased, which is in good agreement with literature reports that a higher AA content causes the hydrogel’s pores to be larger [30]. As such, the pore size of hydrogels could be tuned by adjusting the monomer ratio. Regardless of the mass ratio of the monomers, the porous structures of all hydrogel samples were intact and interconnected, providing good structural stability to the hydrogels. These results demonstrate that the copolymerization of monomers AM, SBMA, and AA via FP yielded a dense polymer network structure for the hydrogel products. Additionally, the densely porous network structure could promote the extensibility of hydrogels, facilitating the rapid response of hydrogels [11,55,56]. This explains the stretchable behavior of the hydrogel in Figure 1d. Hydrogels with porous structures usually show a good ability to swell in water to exhibit good water absorption and swelling capacity [57].

### 2.4. Swelling Behavior of Poly(AM-co-AA-co-SBMA) Hydrogels

**Figure 5:**
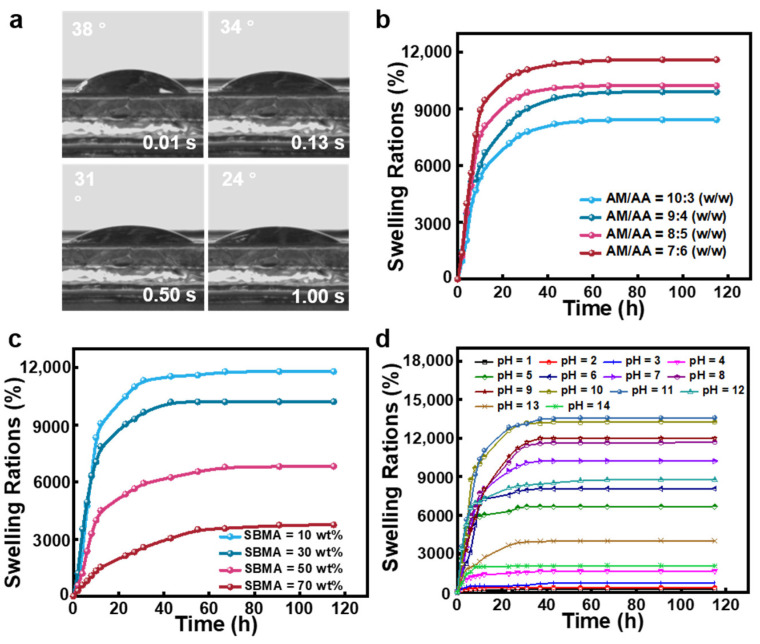
(**a**) Water contact angles of poly(AM-*co*-AA-*co*-SBMA) hydrogels at different residence times. (**b**,**c**) Swelling behavior of poly(AM-*co*-AA-*co*-SBMA) hydrogels prepared with (**b**) different AM:AA mass ratios (SBMA = 10 wt%) and (**c**) different SBMA contents (AM:AA = 7:6 wt/wt). (**d**) Swelling ratios of poly(AM-*co*-AA-*co*-SBMA) hydrogels (AM/AA = 8:5 wt/wt, SBMA = 10 wt%) at different pH values.

First, we investigated the hygroscopic performance of the hydrogels. As seen in Figure 5a, the hydrogel’s contact angle decreased to 24° in 1 s. This feature suggests that the hydrogels are very hydrophilic and have a significant capacity for water absorption. All the AM, AA, and SBMA moieties in poly(AM-*co*-AA-*co*-SBMA) hydrogels have water absorption abilities owing to the presence of abundant hydrophilic groups. Moreover, poly(AM-*co*-AA-*co*-SBMA) hydrogels are composed of three-dimensional cross-linked networks with high porosity and an open pore structure. The high hydrophilicity and flexibility of the polymeric network allow the hydrogels to exhibit a rapid water absorption ability.

Swelling behavior is typical for polymer hydrogels [8]. We investigated the swelling kinetics of poly(AM-*co*-AA-*co*-SBMA) hydrogels synthesized from FP using weight analysis at room temperature with deionized water. In Figure 5b, the hydrogels prepared from different AM/AA mass ratios are shown with their different swelling characteristics. The swelling ratio increased with time for all samples and eventually approached the equilibrium value, i.e., the equilibrium swelling ratio (ESR). Additionally, for the hydrogels synthesized using AM/AA mass ratios of 10:3, 9:4, 8:5, and 7:6 wt/wt, the equilibrium swelling ratios were 8422, 9895, 10,217, and 11,802%, respectively. According to the results, when the AA concentration increased, the equilibrium swelling ratio correspondingly increases. Based on the above results from the characterizations of morphology and swelling behavior, we can conclude that poly(AM-*co*-AA-*co*-SBMA) hydrogels prepared with a higher AA content have larger pore sizes in the cross-linked network structure and higher water absorption and swelling capacities. Figure 5c shows the water swelling ratios of hydrogels with different SBMA contents. As the content of SBMA increased from 10% to 30%, 50%, and 70%, the ESR of the hydrogels decreased from 11,802% to 10,218%, 6820%, and 3746%, respectively. Therefore, poly(AM-*co*-AA-*co*-SBMA) hydrogels have super water absorption capabilities and exhibit large swelling capacities.

The mesh sizes (*ε*) of hydrogels with different monomer mass ratios were further calculated based on the swelling data (see details in the Materials and Methods section) [58,59]. The corresponding mesh sizes were determined to be 59.3, 63.0, 63.6, and 66.7 nm for the hydrogels with AM/AA mass ratios of 10:3, 9:4, 8:5, and 7:6, respectively. Both average pore size from SEM images and mesh size calculated from the swelling data are summarized in Table S1, which can comprehensively describe the microstructure of the hydrogels. Overall, the mesh size of the hydrogels was smaller than the pore size determined from SEM images, consistent with a literature report [60]. In accordance with the SEM results and the swelling results, the smaller the pore size, the smaller the swelling rate and the smaller the value of *ε*, and the denser the network structure of the hydrogel. The swelling behavior of poly(AM-*co*-AA-*co*-SBMA) hydrogels is influenced by the pH value. To investigate the pH-sensitive reactivity of the hydrogels, we submerged the samples in suitable media with a range of pH values (1–14) and measured the swelling characteristics using weight analysis. As shown in Figure 5d, the samples showed good swelling properties in a range of pH solutions. Until the swelling equilibrium was attained, the swelling ratio continuously rose as the swelling time increased. It can be clearly seen that poly(AM-*co*-AA-*co*-SBMA) hydrogels are sensitive to pH. The hydrogels collapsed in the pH range of 1–4, while the equilibrium swelling ratio at pH 6, 7, 8, 9, 10, and 11 were 8066%, 10,218%, 11,683%, 11,991%, 13,253%, 13,588%, and 8766%, respectively. In an alkaline environment, a high swelling ratio is caused by carboxyl group deprotonation and the electrostatic repulsion between groups [61]. However, at pH values of 13 and 14, the equilibrium swelling ratio decreased to 4013% and 2067%, respectively; it is possible that counterions (Na^+^) have a shielding effect that hinders the samples from swelling. The above results demonstrate that these prepared poly(AM-*co*-AA-*co*-SBMA) hydrogels are sensitive to pH.

### 2.5. Rheological Properties of Poly(AM-co-AA-co-SBMA) Hydrogels

**Figure 6:**
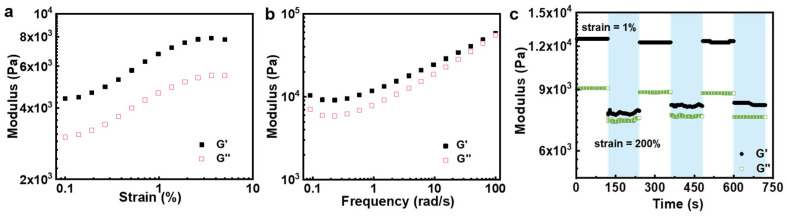
Storage modulus (G′) and loss modulus (G″) of poly(AM-*co*-AA-*co*-SBMA) hydrogels upon (**a**) strain sweeps, (**b**) frequency sweeps, and (**c**) dynamic strain amplitude tests between a strain of 1% and 200%.

We further investigated the rheological properties of poly(AM-*co*-AA-*co*-SBMA) hydrogels using strain sweeps, frequency sweeps, and dynamic strain amplitude tests (Figure 6). First, the solid-like elastic response (storage modulus, G′) and liquid-like viscous response (loss modulus, G″) of the hydrogels were measured with a fixed angular frequency of 1 rad/s and a strain scan mode of 0.1 to 10%. As shown in Figure 6a, the storage modulus G′ of the hydrogels was always larger than the loss modulus G″, indicating that the hydrogels show solid-like behavior in this strain range. Figure 6b shows the frequency scan curves of the hydrogel at a constant strain of 0.1% and a frequency variation range of 0.01–100 rad/s. The hydrogels also maintained a gel state over the entire range of frequencies. Alternate step-strain measurements were then performed on the hydrogels, by applying low (strain = 1%, angular velocity = 1 rad/s) and high (strain = 200%, angular velocity = 1 rad/s) oscillatory strains every 120 s cycle. As shown in Figure 6c, the G′ of the hydrogel was still higher than G″ in all cases, which means that the hydrogels exhibit solid-like behaviors. These results suggest that the hydrogel network is quite stable, allowing the hydrogels to circumvent the collapse of the gel state into the liquid state. Moreover, the hydrogels showed recovery of both moduli G′ and G″ after removing the high strain, revealing the self-recovery ability of the hydrogels after deformation.

### 2.6. Self-Healing Properties of Poly(AM-co-AA-co-SBMA) Hydrogels

**Figure 7:**
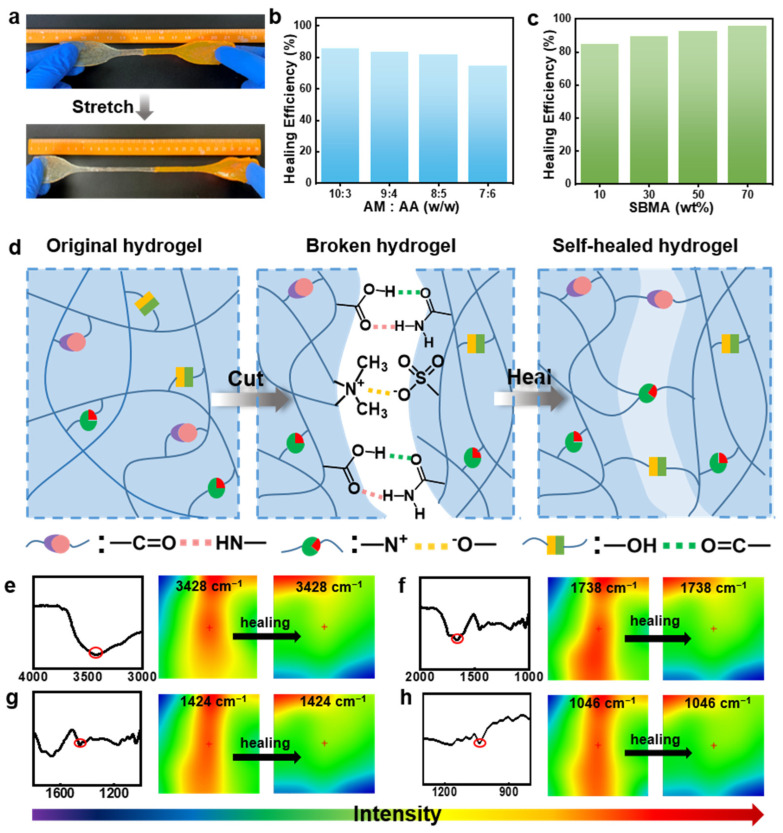
(**a**) Photographs of a healed sample of a poly(AM-*co*-AA-*co*-SBMA) hydrogel under stretching. (**b**,**c**) Self-healing efficiency of poly(AM-*co*-AA-*co*-SBMA) hydrogels prepared with (**b**) different AM/AA mass ratios (SBMA = 10 wt%) and (**c**) different SBMA concentrations (AM/AA = 8:5 (wt/wt), 24 h. (**d**) Self-healing mechanism via ionic association and hydrogen bonding interactions shown in schematic. (**e**–**h**) FT-IR spectroscopy and infrared pictures of these hydrogels before and during self-healing.

Poly(AM-*co*-AA-*co*-SBMA) hydrogels exhibit self-healing properties as well (Figure 7). We prepared two samples of poly(AM-*co*-AA-*co*-SBMA) hydrogels, wherein one sample was stained using rhodamine B to assess their self-healing capability. Subsequently, we cut each sample into two halves, and put together two freshly cut surfaces of stained and unstained hydrogels, which were immediately merged. These hydrogels were then allowed to heal for 24 h at room temperature without any outside stimulation. As seen in Figure 7a, these recovered hydrogels can be strained to great lengths without cracking in the treated area, demonstrating the samples’ excellent capacity for self-healing as well as stretchability. We systematically examined different AM/AA mass ratios and SBMA concentrations to see how they affected the self-healing efficiency (Figure 7b,c). As the AM/AA mass ratio decreased from 10:3 to 7:6 (wt/wt), the self-healing efficiency decreased from 87% to 73%, whereas the healing efficiency varied from 78% to 95% in different hydrogel samples with SBMA mass concentrations ranging from 10 wt% to 70 wt%. These results suggest that a higher AM or SBMA content facilitates the formation of hydrogels with a better healing efficiency. Typically, the dynamic properties and reversibility of non-covalent interactions are meaningful in improving self-healing properties [6,7]. Robust non-covalent interactions including hydrogen bonding and electrostatic interactions exist in poly(AM-*co*-AA-*co*-SBMA) hydrogels that have abundant amide, carboxylic acid, and ester functional groups as well as −N^+^−CH_3_ and −SO_3_^−^ groups. Given that hydrogen bonding interactions and ionic bonding are reversible and can spontaneously recombine after external stimulus breakage [44,54,62,63], we presume that these reversible non-covalent interactions provide the hydrogels with self-healing properties, as shown in the schematic diagram in Figure 7d. When two freshly cut surfaces were put together, hydrogen bonding and electrostatic interactions formed between the dangling functional groups like amide and carboxylic acid, and −N^+^−CH_3_ and −SO_3_^−^, respectively. As such, reversible non-covalent cross-linking occurs between polymer chains, which endows the gel material with a self-healing ability [8,41].

We performed microscopic infrared spectroscopy for the hydrogels to further confirm our proposed mechanism and to elucidate the role of the functional groups. Figure 7e–h display infrared images of the hydrogels at different phases during the process of healing. The diverse colors of the IR pictures represent the corresponding distribution densities of the four different groups: –NH_2_/−OH (3428 cm^−1^), −COOH (1738 cm^−1^), −N^+^−CH_3_ (1324 cm^−1^), and −SO_3_^−^ (1046 cm^−1^); the red color indicates a high concentration as well as blue color indicates a low intensity. These groups are relatively homogeneously distributed in the original sample, which is reflected in the green color in the IR image. After cutting the hydrogel into two parts, abundant functional groups were revealed on the cut surface, exhibiting strong group intensity (colored in red). As the fracture surface gradually healed, the group distribution density weakened as a result of interactions between these groups (including hydrogen bonds between −OH and −COOH or –NH_2_ and electrostatic interactions between −SO_3_^−^ and −N^+^−CH_3_ groups). The uniform green color among the healed region and the virginal region further suggests the high self-healing performance of the hydrogels. These results are consistent with our proposed mechanism that electrostatic interactions and hydrogen bonding act in concert to give the hydrogels excellent self-healing properties.

## 3. Conclusions

In summary, we have rapidly synthesized poly(AM-*co*-SBMA-*co*-AA) superabsorbent and self-healing hydrogels by frontal polymerization within 10 min. The relationships between the chemical components (monomer ratios) of the hydrogels and the kinetics of the frontal polymerization reaction as well as the product properties have been thoroughly investigated. The regulation of the porous structure and equilibrium swelling ratio of the hydrogels could be achieved by varying the monomer ratio. The hydrogels show super water absorption and pH-sensitive swelling behaviors to give a maximum equilibrium swelling ratio of 11,802% in deionized water and 13,588% under alkaline conditions. Rheological studies revealed stable hydrogel networks. The abundant hydrogen bonding and electrostatic interactions in the hydrogel network led to a favorable self-healing ability with a maximum self-healing efficiency of 95%. Thus, the methods and the materials presented herein might provide new insights into the rapid synthesis of hydrogels with excellent swelling capacities and self-healing abilities.

## 4. Materials and Methods

### 4.1. Materials

Acrylic acid (AA), 3-[Dimethyl-[2-(2-methylprop-2-enoyloxy)ethyl]azaniumyl]propane-1-sulfonate (SBMA, 98%), acrylamide (AM), ethylene glycol, Rhodamine B, N,N″-bis(acryloyl)cystamine (BACA, 98%), ammonium persulfate (APS), and N, N, N′, N′-tetramethylethylenediamine (TMEDA) were obtained from Aladdin Industrial Corporation and were used with no additional purification.

### 4.2. Synthesis of Poly(AM-co-AA-co-SBMA) Hydrogels by FP

Poly(AM-*co*-SBMA-*co*-AA) hydrogels were rapidly prepared by a horizontal FP method in the following manner: AM, AA, SBMA, BACA, APS, and ethylene glycol solution were added to a beaker and magnetically stirred to obtain a well-mixed solution. TMEDA reagent was then gradually added to the above solution, stirred, and transferred to a horizontal quartz vessel. To carry out the frontal polymerization reaction, an electric soldering iron set to 100 °C was used to heat the left side of the horizontal quartz vessel. The heat source was removed as soon as a polymerization front formed, allowing the reaction to proceed spontaneously without the need of further energy input. A typical composition of a hydrogel is as follows: AM:AA = 8:5 wt/wt, SBMA = 10 wt%, BACA = 0.5 wt%, APS = 0.2 wt%, ethylene glycol = 50 wt%, and APS/TMEDA = 1:4 mol/mol. All hydrogel samples for characterization required complete immersion in deionized water over the course of seven days with daily water changes to remove unreacted monomers.

### 4.3. Frontal Velocity and Temperature Tests

The frontal polymerization velocity was measured as a function obtained by recording the frontal position over time. Then, plots of frontal position versus reaction time were obtained, where the frontal velocity could be obtained from the slope. Using a FLIR E8 infrared camera, temperature changes over time were recorded at a fixed location to record the temperature profile (a position 4.7 cm from the thermal initiation point was chosen for consideration in this work).

### 4.4. Structural Characterization

A Thermo Nicolet-6700 Fourier Transform Infrared spectrometer was used to take measurements to characterize the chemical structure of the hydrogels. For FT-IR characterization, the samples were completely immerged in deionized water with the water changed daily for 7 days until unreacted monomers and solvents were removed, and the product was dried at 60 °C in a vacuum oven. The treated hydrogel samples were then blended into potassium bromide and powdered. The spectral acquisition was accomplished using 32 scans with a resolution of 4 cm^−1^. The micro-infrared images to analyze the self-healing process was obtained using a Thermo Scientific Nicolet iN10 infrared microscope (Thermo Electron Corporation, Madison, Wisconsin, USA). To carry out the thermogravimetric experiments, a simultaneous thermal analysis instrument (STA 449 F3 Jupiters, Netzsch, Selb, Bavaria, Germany) was heated steadily from 30 to 800 °C at a rate of 10 °C/min.

### 4.5. SEM Measurements

The pore size of the hydrogel was studied using scanning electron microscopy (QUANTA 200, Philips-FEI, North-Brabant, Eindhoven, Holland) at 20.0 kV. The removal of solvents and unreacted monomers in the hydrogels were realized by immersing the hydrogels in pure water for 7 days with daily water changes. The hydrogels reaching the swelling equilibrium were freeze-dried and then cut to expose the internal structure for SEM observation.

### 4.6. Swelling Measurements

The swelling ratio was determined using weight analysis of the hydrogel. Each hydrogel sample tested was initially weighed. Then, the sample was submerged in deionized water and taken out for weighing at regular intervals by removing the excess water from the surface of the sample. This procedure was repeated until the swelling equilibrium was reached. The swelling ratio was calculated using the following equation:

(1) $$\mathrm{Swelling}\;\mathrm{ratio}=\left(W_{t}-W_{0}\right)/W_{0}\times 100\%$$

where *W*_0_ and *W_t_* are the mass of the sample at the initial state and after swelling, respectively.

### 4.7. Mesh Size Based on Swelling Experiment

The mesh size (*ε*) of the poly(AM-*co*-SBMA-*co*-AA) hydrogels was determined using Equations (2) and (3) [58,59]:

(2) $$M_{c}=\frac{n\left(\mathrm{AM}\right)}{n\left(\mathrm{BACA}\right)}M\left(\mathrm{AM}\right)+\frac{n\left(\mathrm{AA}\right)}{n\left(\mathrm{BACA}\right)}M\left(\mathrm{AA}\right)+\frac{n\left(\mathrm{SBMA}\right)}{n\left(\mathrm{BACA}\right)}M\left(\mathrm{SBMA}\right)+M\left(\mathrm{BACA}\right)$$

(3) $$\varepsilon =l{\left(\frac{2M_{c}}{M_{0}}\right)}^{1/2}C_{N}^{1/2}ESR^{1/3}$$

where *M*(AM) is the molar mass of AM (71.08 g/mol); *M*(AA) is the molar mass of AA (72.06 g/mol); *M*(SBMA) is the molar mass of SBMA (279.35 g/mol); *M*(BACA) is the molar mass of BACA (260 g/mol); *n*(AM), *n*(AA), *n*(SBMA), and *n*(BCA) are the moles of AM, AA, SBMA and BACA, respectively; *l* is the length of a C–C single bond (*l* = 0.154 nm); *M*_0_ is the average molar mass of the three monomers and the crosslinker calculated from their proportions in the hydrogel polymer; and *C_N_* is the characteristic ratio, which here, *C_N_* was calculated as 11.46 according to the monomer weight ratio in the hydrogel.

### 4.8. Rheological Characterization

The rheological properties of the hydrogels were evaluated using an Antompa MCR302 at room temperature. The hydrogel had a circular shape with a size of 25 mm in diameter and 3 mm in thickness. Each measurement was performed three times and averaged to check for repeatability.

### 4.9. Self-Healing Measurements

The ability of the hydrogel to self-heal was investigated by performing tensile tests on the healed samples. The ratio of the healed hydrogel in toughness to the toughness of the original hydrogel was considered the healing efficiency of the hydrogel. The SANS CMT6203 tester was used to test the hydrogels at 20 mm/min at room temperature. The samples were made into dumbbell shapes (thickness: 4 mm; width: 6 mm; total length: 11.5 cm; gauge length: 5 cm) which were then cut into two pieces from the middle and adhered together without any other external stimulation. The samples were allowed to heal for 24 h at room temperature, and tensile tests were performed on the original and the healed samples. Each test was performed three times, and the average data from these calculations were used to determine the self-healing efficiency.

## Data Availability

The data presented in this study are available on request from the corresponding author.

## Supplementary Materials

The following supporting information can be downloaded at: https://www.mdpi.com/article/10.3390/gels9050380/s1, Figure S1. Pictures of (a) the setup for the horizontal FP process and (b) self-propagating front during the process of FP enabling the transformation of monomers to hydrogel polymers of poly(AM-*co*-AA-*co*-SBMA); Figure S2. Pore size distributions of poly(AM-*co*-AA-*co*-SBMA) hydrogels synthesized from AM/AA ratios of (a) 10:3, (b) 9:4, (c) 8:5, and (d) 7:6 (wt/wt). SBMA = 10 wt%; Table S1. Average pore size and mesh size of poly(AM-*co*-AA-*co*-SBMA) hydrogels.
