# Supplementary material for: Rapid Preparation of Superabsorbent Self-Healing Hydrogels by Frontal Polymerization
Source: Gels. 2023 May 5;9(5):380. doi: 10.3390/gels9050380 (PMC10217271; doi:10.3390/gels9050380)
Supplement: Supplementary file 1 [file gels-09-00380-s001.zip › gels-2363708-supplementary.pdf]

Supplementary Materials

Rapid Preparation of Superabsorbent Self-Healing Hydrogels by Frontal Polymerization

Ying Qin, Hao Li, Hai-Xia Shen, Cai-Feng Wang,* and Su Chen*

State Key Laboratory of Materials-Oriented Chemical Engineering, College of Chemical Engineering, Nanjing Tech University, 5 Xin Mofan Road, Nanjing 210009, P. R. China.
Correspondence: caifengwang@njtech.edu.cn; chensu@njtech.edu.cn.

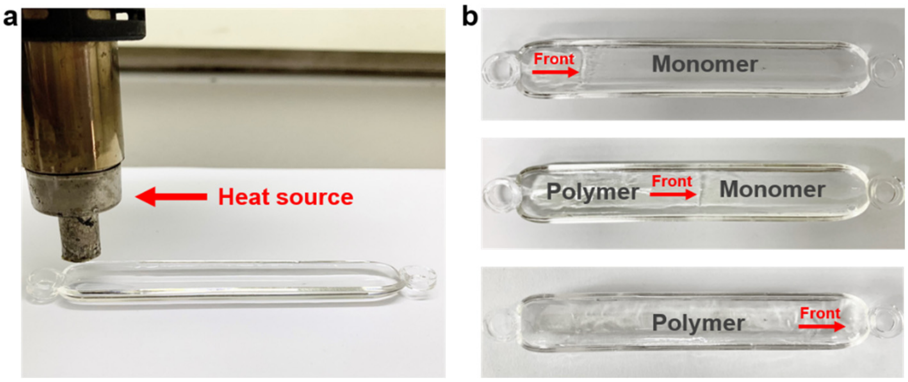

Figure S1. Pictures of (a) the setup for the horizontal FP process and (b) self-propagating front during the process of FP enabling the transformation of monomers to hydrogel polymers of poly(AM-co-AA-co-SBMA).

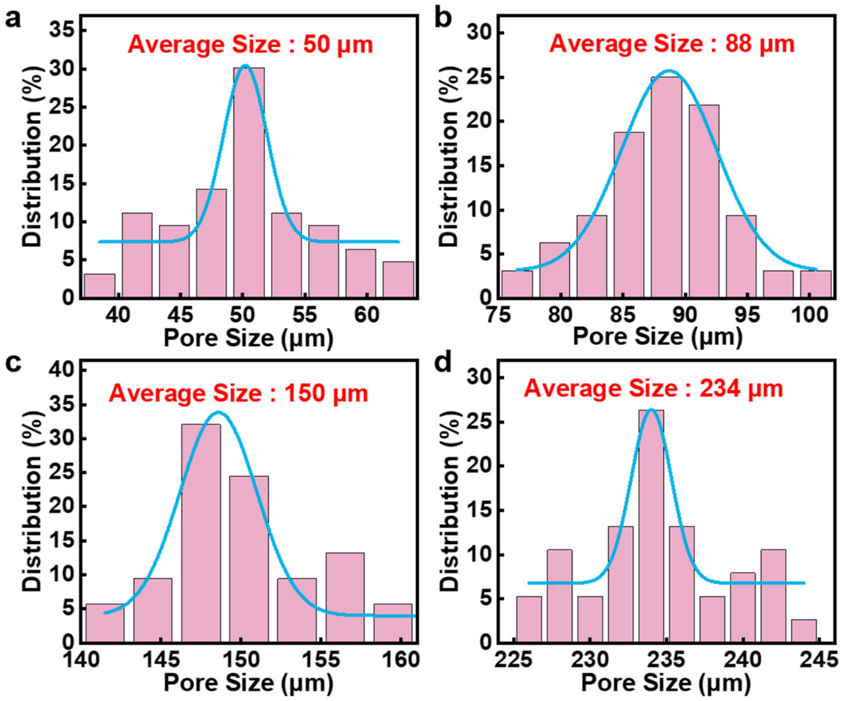

Figure S2. Pore size distributions of poly(AM-*co*-AA-*co*-SBMA) hydrogels synthesized from AM/AA ratio of (a) 10: 3, (b) 9: 4, (c) 8: 5 and (d) 7: 6 (wt/wt). SBMA = 10 wt%.

Table S1. Average pore size and mesh size of poly(AM-co-AA-co-SBMA) hydrogels.

AM/AA mass ratio (wt/wt)	Average pore size (μm)	Mesh size (nm)
10: 3,	50	59.3
9: 4	88	63.0
8: 5	150	63.6
7: 6	*234*	66.7
